# Biomarkers in Aneurysmatic and Spontaneous Subarachnoid Haemorrhage: A Clinical Prospective Multicentre Biomarker Panel Study of S100B, Claudin-5, Interleukin-10, TREM-1, TREM-2 and Neurofilament Light Chain As Well As Immunoglobulin G and M

**DOI:** 10.1007/s12035-025-04889-3

**Published:** 2025-04-28

**Authors:** Thomas Kapapa, Andreas Pfnür, Rebecca Halbgebauer, Patrik Broer, Steffen Halbgebauer, Hayrettin Tumani, Ann-Kathrin Friedrichs, Markus Huber-Lang, Lena Dörfer

**Affiliations:** 1 Department of Neurosurgery, University Hospital Ulm, Albert-Einstein-Allee 23, Ulm, 89081 Germany; 2 Institute of Clinical and Experimental Trauma Immunology, University Hospital Ulm, Helmholtzstraße 8/1, Ulm, 89081 Germany; 3 Department of Intensive Care Medicine, Hospital Winterthur, Brauerstrasse 15, Winterthur, 8401 Austria; 4 Department of Neurology, University Hospital Ulm, Oberer Eselsberg 45, Ulm, 89081 Germany

**Keywords:** Delayed cerebral ischemia, Secondary brain injury, Vasospasm, Prognosis

## Abstract

Following aneurysmatic subarachnoid haemorrhage (SAH), complex pathophysiological processes take place which result in ischaemia, dysfunction of the blood–brain barrier and the clinical development of vasospasms and delayed cerebral ischaemia (DCI). The aim of this study was to present a biomarker panel that can be used for temporal assignment in the pathophysiological process after haemorrhage, a prediction of vasospasm, DCI or outcome. In a prospective multicentre approach, complex laboratory chemistry tests were used to determine the value of the biomarkers S100B, Claudin-5, Interleukin (IL) -10, Triggering receptor expresses on myeloid cells (TREM)-1 and TREM-2, and neurofilament light chain (NfL) as well as IgG and IgM in plasma and Cerebro-spinal-fluid (CSF) in SAH patients. The predictive power of mentioned biomarkers with regard to the occurrence of vasospasms, DCI and the outcome (Glasgow Outcome Scale) were defined by using sophisticated statistical methods with the level of significance at *p* ≤ 0.05. Mean age of the 12 patients included was 56 (SD:14) years with 67% female patients and that of the 11 control subjects was 74 (SD:3) years with 55% female subjects. S100B showed higher concentrations compared to the control patients on the first four days (*p* ≤ 0.0141). For IL-10, the CSF concentrations showed a continuous increase: day 2 (*p* = 0.0074), day 4 (*p* = 0.0012), and day 5 (*p* < 0.0001). Regarding the TREM1 and TREM2 balance, CSF concentrations of TREM1 increased until day eight (*p* ≤ 0.0055). TREM-2 plasma concentrations decreased below the levels of control patients and appeared unchanged for the further course. The greatest difference in the CSF concentration of NfL between the patients and the control group was seen on day 8 (*p* = 0.0104). The differentiation between patients with and without DCI showed different concentration curves of the TREM1 CSF-plasma index with increasing concentrations for patients with DCI. The TREM 2 CSF-plasma index showed higher concentrations for patients with DCI. Patients without DCI showed a decreasing concentration of the NfL CSF-plasma index compared to an increase when vasospasm was detected. NfL, TREM-1 and TREM-2 have the potential to be relevant biomarkers for SAH in the intermediate and delayed injury phase.

**Supplementary Information:**

The online version contains supplementary material available at 10.1007/s12035-025-04889-3.

## Background

Aneurysmatic (non-traumatic) spontaneous subarachnoid haemorrhage (SAH) is a pathological condition characterised by high morbidity (poor outcome modified Rankin Scale 4 to 6 in 28%) and mortality (pre-hospital 26%, in-hospital 20%), including a life-threatening form of stroke [1, 2]. In most of the cases (> 80%), the origin is the rupture of an aneurysm in the intracranial arteries supplying the brain [3]. Whereas in traumatic brain injury (TBI), biomarker profiles have been intensively investigated to assess the posttraumatic immuno-pathophysiological response [4], less is established along the dynamic axis of SAH.

The complex post-haemorrhagic processes can be divided into a phase of early brain injury with acute ischemia (days 0–3), a phase of intermediate injury (days 4–5) and a phase of delayed injury (days 6–14) [5]. The first phase of early brain injury is characterized by the translocation of blood cells and blood components including erythrocytes, haemoglobin, fibrin and fibrinogen into the cerebro-spinal-fluid (CSF). The degradation may lead to free radicals and hydrogen peroxides, which build up an autoregressive stress [5]. In addition, changes in the blood–brain barrier (BBB) occur due to enzymatic degradation and mechanical stress on the glycocalyx, which assembles an important inner layer of the inner vessel wall [6, 7]. These changes facilitate the development of neuroinflammation including immigration of inflammation-competent cells into the CSF and central nervous system (CNS) [7–9]. In this phase, neuroinflammation manifests through microglia activation and monocyte recruitment, resulting in the secretion of interleukins [10], along with astrocyte activation marked by neurotoxicity and upregulation of S100B [11, 12]. In the subsequent intermediate phase, dysfunction of endothelial cells and pericytes ensues [13], leading to an increased influx of immunocompetent cells and the activation of pericytes, thereby perpetuating inflammatory processes [5]. The third and final phase is the phase of delayed injury, which is characterized by a progressive destruction of gap junctions between the astrocytes that leads to astrocytic apoptosis [14]. The additional loss of connections between neurons and astroglia is associated with a disruption of the energy balance of the neurons, with the consequence of a disturbed cellular sodium–potassium-pump [15, 16], with subsequent spreading depolarization [17]. The altered and further disturbed cell metabolism and energy balance leads to an increase in neuroinflammatory signalling (cytokines/chemokines such as interleukins (IL) and inflammatory cells such as regulatory and cytotoxic cells) and to increased cell death [18, 19].

Based on these inter- and intracellular processes, current scientific and clinical endeavours focus on identifying metabolic or cellular markers that enable prediction of the further clinical course and complications [20]. Regarding SAH, these biomarkers should appear primarily in the CNS or in the CSF, and should be absent or in low concentration in the peripheral blood (PB) [21]. Since the pathophysiological processes, as indicated, are very complex in their timing and in terms of cellular metabolism, it is unlikely to find a single biomarker that meets the requirements of transcriptomic, cellular (cell damage), inflammatory, metabolic, cerebro-vascular and clinical prediction [22]. Therefore, a more plausible approach involves identifying a panel of biomarkers that is anticipated to exhibit greater predictive potency than a solitary biomarker [22]. The objective of our clinical study is therefore to present various spatial-specific biomarkers in the pathological condition of SAH. For our observational approach, the biomarkers S100B, IgG, IgM, Claudin-5, TREM-1, TREM-2, neurofilament light chain (NfL) and IL-10 were selected. Claudin-5 to some extend correlates with the integrity and function of the BBB sealing endothelial cells in the vessels supplying the brain and being expressed on astrocytes and pericytes as bicellular tight-junctions [23–28]. TREM-1 plays an essential role in the receptor pathways of microglia-mediated inflammation after SAH and thus also contribute to the BBB function [29–31]. TREM-2 plays a role in microglia-mediated inflammation and axonal injury [32, 33]. NfL is a sensitive but non-specific marker for axonal damage and degeneration [34]. IL10 is a well-known and broadly used biomarker for assessing the outcome after infectious and inflammatory processes [35–38]. S100B from the S100 family is a well-known biomarker for astrocyte-mediated CNS inflammation, cellular homeostasis and neuronal apoptosis as well as integrity of the BBB [39–42, 42]. Clinically, it is used as a outcome predictor in acute brain injury [42–44]. S100B was selected as a control marker. IgG and IgM were analysed as indicators for CNS infections. We aim to discern their specificity for CNS damage in context of vasospasms or delayed cerebral ischemia (DCI), detectable in the CSF and less evident in the PB.

## Methods

### Study Design

The study presented was a prospective, two-centre, observational patient study. Patients who were consecutively treated for SAH in the two hospitals between 2008 and 2015 were included. The two neurosurgery departments are embedded in a tertiary care concept and have access to a neurocritical care ward. The authors found no difference in the treatment of patients, as the treatment algorithms are based on current national and international guidelines [1]. The study was carried out after several approvals from the independent local ethics committee of the University of Ulm (No. 82/07, 31st July 2007; No. 157/20, 13th July 2020). Written informed consent for inclusion in the study was obtained from the patients or their legal representatives. Within the first 6 h after the haemorrhage and then in six hour intervals, 5 to 10 ml CSF as well as PB were collected. This continued until vasospasm or DCI occurred and 24 h beyond this or, without the occurrence of vasospasm or DCI, up to the 11th day after SAH. The primary endpoint was the angiographic or clinical evidence of vasospasm or a DCI. The secondary endpoint was the clinical outcome after 12 months. The aim was to identify biomarkers being valid for predicting vasospasm or DCI in relation to the outcome or to describe the temporal aspects of molecular pathophysiology after SAH.

### Patient’s Study Population, Standard Operating Procedures

Patients aged ≥ 18 years (*n* = 11), with aneurysmatic SAH according to hospital diagnostic standards and requiring ventricular drainage for initial acute hydrocephalus were included. One patient had the cardinal symptoms and pathgnomonic computed tomographic intracranial blood distribution of aneurysmal SAH, which is why we performed the inclusion within 6 h of haemorrhage. At the time of sampling as well as afterwards, the treatment team assumed that an aneurysm would be diagnosed in the further course. Unfortunately, the patient refused further diagnostic steps in the course of the procedure, meaning that an aneurysm could not be ruled out with certainty. Hydrocephalus was defined by the bicaudate index > 0.16 [45]. The standard operating procedures (SOPs) of the two departments for neurosurgery include: SAH was confirmed using computed tomography (CT), magnetic resonance imaging (MRI) or lumbar puncture. Clinical severity was graded according to the World Federation of Neurosurgical Societies (WFNS) Scale [46, 47]. Acute hydrocephalus as increase in the ventricular size (bicaudate index > 0.16 (> 95th) percentile for age) was treated by external ventricular drainage [1, 45, 48]. For inclusion, the diagnosis and the placement of the ventricular drainage within the first 6 h after haemorrhage or first symptoms was mandatory. The severity of bleeding was graded using modified Fisher’s scale [49, 50]. Aneurysms should be sealed within 24 h after bleeding. The type of aneurysm sealing (endovascular or surgical) is discussed and decided on an interdisciplinary basis between neuroradiologists and neurosurgeons. After the acute treatment (emergency resuscitation, intensive care stabilization, implantation of the ventricular drainage and aneurysm sealing), neuro-intensive care treatment took place. Daily transcranial doppler ultrasound (TCD) was performed to detect vasospasm. In the event of clinical deterioration, diagnostic imaging to clarify DCI was carried out using imaging (digital subtraction angiography, CT angiography and perfusion scanning). Clinical examinations were carried out twice daily with grading the Glasgow Coma Scale (GCS) or Brussels Coma Scale (BCS) [51, 52]. Patients routinely received oral nimodipine at a dosage of 60 mg every 4 h, and, if necessary, venous or continuous intra-arterial administration of nimodipine after evidence of DCI according to clinical severity [1, 53]. In order to detect a systemic infection, daily blood checks of the C-reactive protein and blood count checks were carried out. To detect a CSF infection or meningitis, the CSF was analysed at regular intervals using laboratory biochemistry and microbiology tests. The definition of a CSF infection or meningitis followed the current guidelines and definitions with an increased cell count, decreased glucose content and increased lactate content with a predominant presence of neutrophilic cells outside the reference ranges [54]. A CSF infection or meningitis was definitely present with microscopic or microbiological evidence of pathogenic germs in the CSF. Antibiotic therapies were only initiated in the case of CSF laboratory values corresponding to a CSF infection or meningitis and/or if pathogens were detected in the CSF. Following exclusion criteria were applied: the occurrence of previous illnesses such as alcohol and drug abuse, vascular diseases, genetic disorders affecting the connective tissue, cancer, degenerative, acute or chronic (inflammatory) CNS diseases, coagulation disorders, acute infections, a life expectancy < 96 h, and other surgical interventions on the CNS (e.g. decompressive craniectomy).

### Control Population

Control CSF and serum samples analysed in this study were from patients seen at a department of Neurology between 2014 and 2020. The patients presented themselves on the basis of various symptoms. None of the patients had an intracranial or spinal haemorrhage, which is why they were identified as control patients. Nevertheless, there was a clinical need for clarification of the CSF and thus for a lumbar puncture. The diagnoses of the control patients are shown in supplementary Table 1. The study was approved by the independent local ethics committee of the University of Ulm (No. 20/10, 3rd May 2010 and 2nd June 2021) and conducted following the Declaration of Helsinki. All participants gave their written informed consent to participate in the study. The control patients demonstrated no clinical or radiological evidence of an underlying neurodegenerative or neuroinflammatory disease. For all patients, a lumbar puncture was performed to rule out an acute or chronic inflammation of the CNS. The CSF of the control population is therefore lumbar CSF in contrast to the patients whose CSF is of ventricular origin. There is a considerable difference in the composition of lumbar and ventricular CSF [55]. However, surgery to place an external ventricular drain in control patients does not have a positive risk–benefit ratio [56]. Therefore, we did not perform ventricular CSF sampling in the healthy control population in our study.

### Clinical Definitions

The clinical definition of Delayed Cerebral Ischemia (DCI) is “The occurrence of focal neurological impairment (such as hemiparesis, aphasia, apraxia, hemianopia or neglect), or a decrease of at least 2 points on the Glasgow Coma Scale, (either on the total score or on one of its individual components [eye, motor on either side, verbal]). This should last for at least 1 h, is not apparent immediately after aneurysm occlusion, and cannot be attributed to other causes by means of clinical assessment, CT or MRI scanning of the brain, and appropriate laboratory studies” [57]. Cerebral vasospasm can be defined and subdivided into an angiographic vasospasm (“moderate-to-severe arterial narrowing on digital subtraction angiography not attributable to atherosclerosis, catheter-induced spasm or vessel hypoplasia, as determined by a neuroradiologist”) and TCD-detected disturbances of blood flow (i.e. mean flow velocity in any vessel > 120 cm/sec) [58]. Angiographic vasospasm can be more specific defined as “a decrease in diameter of greater than 30% compared with the adjacent contiguous vessel segment” (mild 10–30%, moderate 30–50% and severe > 50%) [59].

### Sampling and Laboratory Analysis

Under strict sterile conditions, 5 to 10 ml of CSF were taken from the ventricular drain system and collected in colourless sterile polypropylene tubes (Sarstedt, Germany). PB (7.5 ml) was taken from an existing arterial access and carried out in a timely manner after the CSF collection (Serum Gel and Plasma Gel with Lithium-Heparin tubes from Sarstedt, Germany). The collected samples were processed expeditiously to prevent later interferences (e.g., cell degradation or metabolic effects). The CSF was centrifuged at 1000 rpm and the PB at 3600 rpm for 10 min. The cell-free CSF and the plasma was pipetted in tubes, marked with study details and stored at minus 80 °C. The corresponding mean concentrations of biomarkers were determined in both, CSF and plasma (serum in control patients), as well as the respective de novo synthesis rate was calculated and put in relation to the primary and secondary endpoints. IgG quotations and derivates in the CSF (IgG Index > 0.7) were considered indicative of CNS infections or inflammatory processes [60, 61]. The same applied to IgM [62].

#### ELISA

All ELISAs were performed according to the manufacturer’s instructions. The human IgM ELISA kit (ab137982, Abcam, UK) and the human IgG ELISA kit (ab195215, Abcam, UK) were used to measure the immunoglobulin levels in plasma (serum in control patients) and CSF samples. Concentrations of TREM-1 (DY1278B, R&D systems, USA), TREM-2 (DY1828-05, R&D systems, USA), IL-10 (DY217B, R&D systems, USA), S100B (DY1820-05, R&D systems, USA), Claudin-5 (CSB-EL005507HU, CUSABIO Biotech; USA) and NfL (Simple Plex Human NF-L Cartridge, bio-techne, Minneapolis, USA) were determined in plasma (serum in control patients) and CSF strictly in accordance to corresponding manufacturer’s protocol.

### Data Sources and Outcome Variables

Clinical variables recorded are represented by artificial ventilation (hours), vital parameters (heart rate, mean arterial pressure), type and time of diagnostic imaging findings (CT, MRI, digital subtraction angiography = DSA), WFNS Scale, Fishers’ Grade, GCS, BCS (during the course and at discharge), neurological findings during the course (motor function, cranial nerve findings), vascular risk factors, documented previous illnesses, aneurysm location, size and configuration and type of treatment (surgical vs. endovascular), occurrence and duration of vasospasm and DCI (clinical symptoms, TCD, CT and DSA results) and treatment (conservative vs. invasive), occurrence of complications during the course including infarcts, thrombosis and embolism. Outcome was determined by the Glasgow Outcome Scale (GOS) at discharge, after 6 and 12 months [63]. The five-stage GOS was dichotomized and divided into favourable outcome (GOS IV and V) and unfavourable outcome (GOS I to III). Due to the small number of patients in this study, we did not use the sliding dichotomisation approach [64]. Healthcare-associated infections occur in critical care of patients with neurological diseases. Regular monitoring of systemic infection and inflammation parameters such as C-reactive protein (CRP, mg/l) and the number of leucocytes (N/µl) was therefore carried out. The parallel monitoring of these parameters has a direct influence on the concentration of biomarkers that are strongly related to inflammatory processes [65]. As compromised coagulation also has an influence on the outcome after SAH, the values Quick (percent), partial thromboplastin time (PTT, seconds) and the number of platelets (N/µl) were recorded to monitor coagulation during the course [66].

### Statistical Analyses (Tests, Interpretation)

The statistical evaluation and analysis was carried out with the assistance of the local Institute for Epidemiology and Medical Biometry, University of Ulm. To describe quantitative parameters, categorizations such as mean, standard deviation, median and range were used. Qualitative variables were calculated using absolute and relative frequencies. The results were presented in descriptive statistics and corresponding graphs. The target variable were the occurrence of symptomatic vasospasm, DCI and the GOS after 12 months. The influencing variables were primarily the biomarker concentration in CSF and PB as well as their indexes. The categorical variables were calculated using either Fishers’ test or chi-square test. Continuous variables were calculated with non-parametric test. The level of significance was set to *p* ≤ 0.05.

## Results

Twelve patients and eleven control patients were included. The mean age of the patients was 56.7 (SD:13.5) and of the control patients was 73.7 (SD:3.2). There were 8 (67%) female patients and 6 (55%) female control patients. Eleven (92%) patients underwent endovascular sealing and one (8%) patient had no detectable aneurysm. The majority of patients had WFNS grade V (*N* = 6, 50%) and Fisher grade IV (*N* = 7, 58%). Five (42%) patients had vasospasm, 8 (67%) patients experienced a DCI mostly at day 9 (SD: 2.4). The characteristics of the patients are shown in Table 1.

**Table 1:** The characteristics of the patients

	Patients (***N*** = 12)	Controls (***N*** = 11)	Angiographic vasospasm		***p***	Delayed cerebral ischemia		***p***
			Positive (***N*** = 5)	Negative (***N*** = 7)		Positive (***N*** = 8)	Negative (***N*** = 4)	
**Age** (mean, SD)	56.7 (13.5)	73.7 (3.2)	53.2 (13.2)	59.1 (14.2)	0.222	55.1 (14.5)	59.8 (12.6)	0.234
**Female patients** (*N*, %)	8 (67)	6 (55)	3 (60)	5 (71)	1.0	5 (63)	3 (75)	1.0
**Rate of hypertension** (*N*, %)	5 (42)		3 (60)	2 (29)	0.558	4 (50)	1 (25)	0.576
**Rate of diabetes** (*N*, %)	2 (17)		2 (40)	0 (0)	0.152	2 (0)	0 (0)	0.515
**Rate of smoking** (*N*, %)	3 (25)		2 (40)	1 (14)	0.532	2 (25)	1 (25)	1.0
**Aneurys location**								
Anterior cerebral artery (*N*, %)	1 (8)		0 (0)	1 (14)	0.671	0 (0)	1 (25)	0.110
Anterior communicatin artery (*N*, %)	5 (42)		3 (60)	2 (29)		4 (50)	1 (25)	
Middle cerebral artery (*N*, %)	1 (8)		1 (20)	0 (0)		1 (12.5)	0 (0)	
Internal carotide artery (*N*, %)	3 (25)		1 (20)	2 (29)		2 (25)	1 (25)	
Vertebrobasilar artery *N*, %)	1 (8)		0 (0)	1 (14)		1 (12.5)	0 (0)	
No aneurysm (*N*, %)	0 (0)		0 (0)	1 (14)		0 (0)	1 (25)	
**WFNS score (*N*, %)**								
Grade I	1 (8)		0 (0)	1 (14)	0.600	1 (12.5)	0 (0)	0.855
Grade II	1 (8)		1 (20)	0 (0)		1 (12.5)	0 (0)	
Grade III	1 (8)		0 (0)	1 (14)		0 (0)	1 (25)	
Grade IV	3 (25)		1 (20)	2 (29)		2 (25)	1 (25)	
Grade V	6 (50)		3 (60)	3 (43)		4 (50)	2 (50)	
**Modified Fisher grade (*N*,%)**								
Grade I	0 (0)		0 (0)	0 (0)	0.647	0 (0)	0 (0)	0.632
Grade II	2 (17)		0 (0)	2 (29)		1 (12.5)	1 (25)	
Grade III	3 (25)		2 (40)	1 (14)		2 (25)	1 (25)	
Grade IV	7 (58)		3 (60)	4 (57)		5 (62.5)	2 (50)	
**Glasgow Coma Scale**								
Mean (SD)	7.4 (4.5)		6.4 (4.9)	8.1 (4.5)	0.408	7.6 (4.84)	7.0 (4.55)	0.862
Median (range)	7 (3–15)		3 (3–14)	8 (3–15)		7 (3–15)	6 (3–13)	
**Duration of artificial ventilation (in days)**								
Mean (SD),	8.3 (7.9)		11.6 (9.5)	5.9 (6.3)	0.282	9 (8.75)	6.8 (7.14)	1.0
Median (Range)	4.5 (1–24)		14 (1–24)	3 (1–17)		8 (1–24)	5 (1–17)	
**Rate of DCI (*N*, %)**	8 (67)							
Mean days to occurrence (SD)	9 (2.4)		9.4 (1.8)	7.3 (3.1)	0.365	8.6 (2.39)	N.A	
Median days to occurrence (range)	9 (5–12)		9 (7–12)	8 (4–10)		9 (4–12)	N.A	
**Ischemic lesion in imaging (*N*,%)**	9 (75)		4 (80)	5 (71)	1.0	7 (87.5)	2 (50)	0.176
**Glasgow Outcome Scale (GOS) after 12 months (*N*, %)**								
Grade I	4 (33)		1 (20)	3 (43)	0.729	3 (37.5)	1 (25)	0.651
Grade II	3 (25)		2 (40)	1 (14)		2 (25)	1 (25)	
Grade III	0 (0)		0 (0)	0 (0)		0 (0)	0 (0)	
Grade IV	0 (0)		0 (0)	0 (0)		0 (0)	0 (0)	
Grade V	5 (42)		2 (40)	3 (43)		3 (37.5)	2 (50)	
**Unfavourable outcome after 12 months (GOS I-III; *N*, %)**	7 (58)		3 (60)	4 (57)	1.0	5 (62.5)	2 (50)	1.0

There were increases in systemic inflammatory parameters like CRP and the leucocytes count in PB. CRP increases significantly from a baseline value within the reference range (< 5.0 mg/l) from day 1 to 10 (*p* ≤ 0.0002). On day 13 there is a decrease of the CRP concentration. There were no significant changes in the leucocytes count in the PB in the first 13 days after SAH. There were also no significant differences due to the occurrence of vasospasm or DCI. The coagulation parameters Quick and PTT showed no significant differences in the course or in the differentiation between patients with and without vasospasm or DCI. The platelets count in the PB showed a non-significant increase from day 1 to day 10. Only the count day 1 to day 13 showed a significant difference (*p* = 0.0022) (Table 2). Within the first 13 days, CSF samples were regularly sent for microbiological determination. No contamination with pathogens was detected in any patient.

**Table 2:** Significant difference on days 1 to 13

	On admission						Day 2 to 4						Day 8					
	Mean (SD)	Angiographic Vasospasm		Delayed Cerebral Ischemia		P	Mean (SD)	Angiographic Vasospasm		Delayed Cerebral Ischemia		P	Mean (SD)	Angiographic Vasospasm		Delayed Cerebral Ischemia		P
		pos.	neg.	pos.	neg.			pos.	neg.	pos.	neg.			pos.	neg.	pos.	neg.	
Serum C-reactive protein (mg/L)	3.65 (2.55)	4.62 (3.50)	2.96 (1.56)	4.00 (3.00)	2.95 (1.37)	n.s.s.	65.04 (53.87)	93.82 (65.62)	44.48 (35.57)	67.99 (61.77)	59.14 (40.84)	n.s.s.	81.77 (57,39)	98.86 (53.92)	64.68 (61.43)	84.31 (54.26)	75.83 (76.91)	n.s.s.
Serum Leucocytes (N/µl)	13.13 (5.02)	12.34 (4.27)	13.69 (5.75)	13.99 (4.68)	11.40 (5.93)		10.65 (3.46)	11.07 (4.70)	10.34 (2.63)	11.01 (3.93)	9.93 (2.60)		12.48 (2.73)	12.32 (2.42)	12.64 (3.29)	13.34 (2.69)	10.47 (1.76)	
Quick (%)	94.6 (17.08)	97.6 (15.90)	92.4 (18.80)	92.0 (17.93)	99.8 (16.32)		96.6 (8.21)	95.8 (5.74)	97.2 (10.03)	94.6 (9.11)	100.7 (4.54)		102.7 (12.79)	93.5 (12.61)	110.0 (7.51)	99.8 (13.88)	108.3 (10.02)	
PTT (sec)	29.0 (4.49)	29.6 (5.13)	28.6 (4.35)	29.0 (4.84)	29.0 (4.40)		33.7 (4.67)	34.2 (4.11)	33.4 (5.34)	33.5 (3.57)	34.2 (7.07)		31.7 (7.68)	29.8 (3.59)	33.2 (10.08)	32.5 (9.14)	30.0 (4.58)	
Platelet Count (N/µl)	219.3 (71.58)	201.8 (60.28)	231.9 (80.82)	225.9 (69.63)	206.3 (84.45)		195.2 (72.16)	178.2 (47.28)	207.4 (87.41)	205.6 (74.92)	174.4 (71.61)		245.3 (90.37)	230.8 (76.58)	259.8 (109.47)	270.9 (97.79)	185.67 (21.01)	

***    p = 0.0002, **p = 0.0022, **** p < 0.0001

**Fig. 1:**
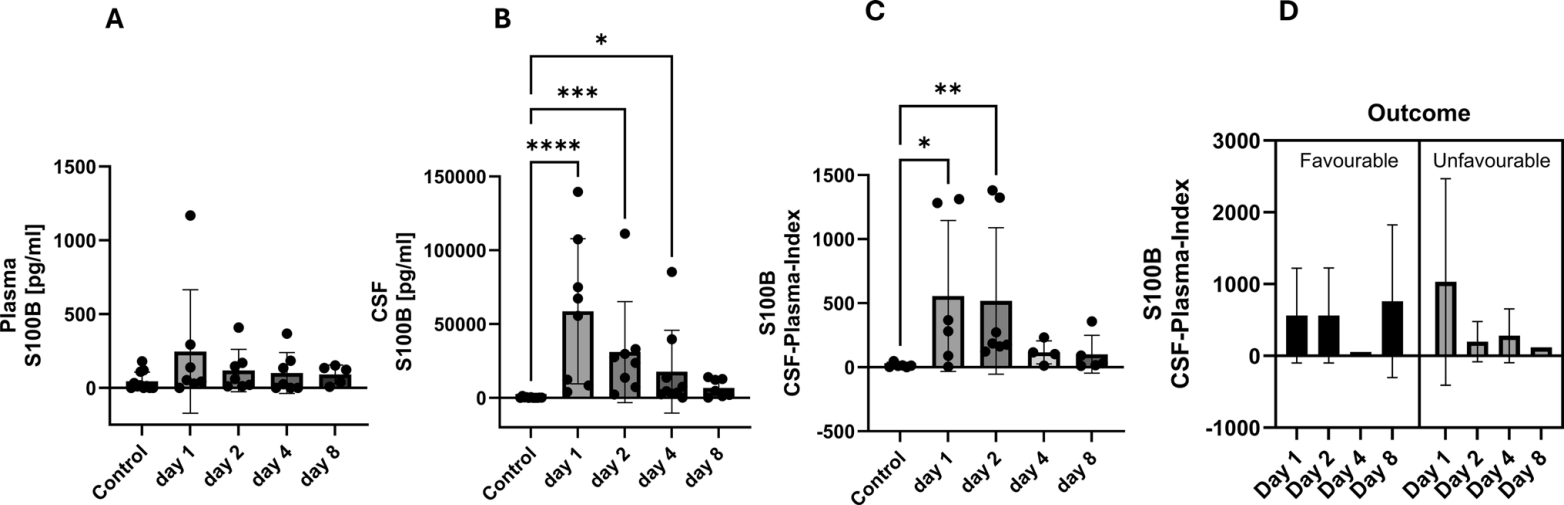
Plasma and cerebrospinal fluid (CSF) concentrations of S100B over the course of the study over 8 days. The control patients have an initial one-time value in the serum. The CSF plasma quotient is used to assess the blood–brain (CSF) barrier function (quotient = CSF concentration / plasma (serum) concentration). S100B appears as an early phase biomarker. The predictive power of S100B for the outcome shows no significant results

Early after SAH, S100B was slightly elevated in plasma while in CSF there was a significant increase during the early and intermediate phase returning to baseline at day 8 post haemorrhage. S100B values revealed significantly higher concentrations compared to the healthy controls on the first day (*p* =  < 0.0001, Kruskal–Wallis test), on the second day (*p* = 0.0002, Kruskal–Wallis test) and on the third day (*p* = 0.0141, Kruskal–Wallis test) (Fig. 1A, B). The CSF-plasma index for S100B confirmed the course with a significant difference to the control patients on the first (*p* = 0.0223, Kruskal–Wallis test) and second day (*p* = 0.0032, Kruskal–Wallis test) after haemorrhage (Fig. 1C). Regarding the outcome, there was a non-significant trend of decreasing values of the CSF-plasma index in patients with unfavourable outcome compared to rather constant values (exception day 4) in patients with favourable outcome, excluding day 4 (Fig. 1D).

**Fig. 2:**
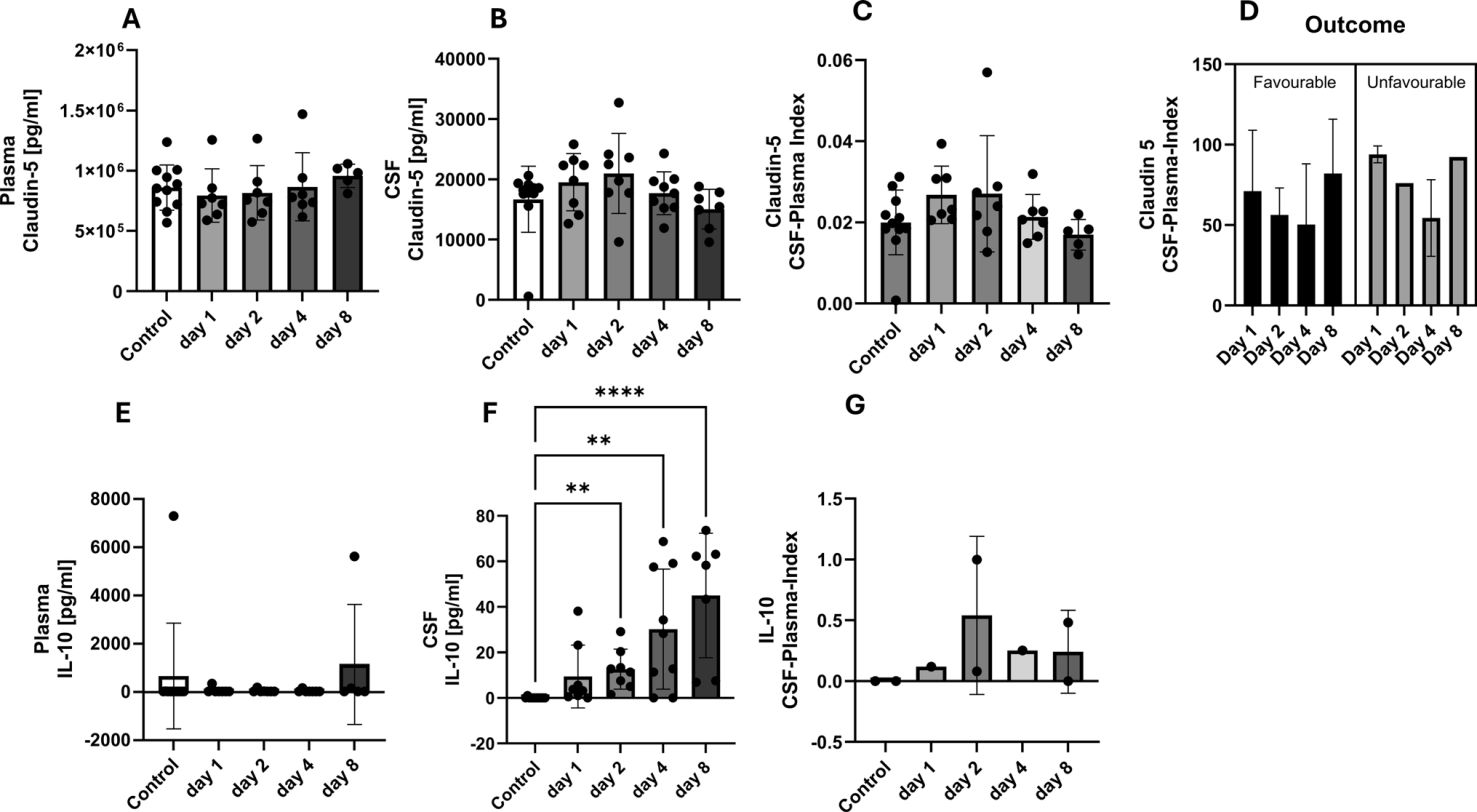
The plasma and cerebrospinal fluid (CSF) concentrations of claudin-5 and IL-10 were measured over the course of the study, which spanned 8 days. The control patients each had an initial one-time value in the serum, and the CSF plasma quotient was used to assess the blood–brain (CSF) barrier function (quotient = CSF concentration / plasma (serum) concentration). Claudin-5 appeared as an intermediate phase biomarker, and IL-10 as a late phase biomarker. The predictive power of claudin-5 and IL-10 for the outcome shows no significant results

The tight junction marker claudin-5 concentrations, in plasma, CSF and CSF-plasma index, increased in all by trend during the intermediate phase with an enhanced variance of the values, reaching baseline levels day 8 after SAH (Fig. 2A, B, C). In terms of outcome, patients with a favourable and unfavourable outcome did not differ with regard to the course of the claudin 5 CSF-plasma index. After a decrease in concentrations from day 1 to day 4, there was an increase on day 8 in both patient groups. It was not possible to differentiate the outcome using claudin 5 as a biomarker (Fig. 2D).

IL-10 revealed a consistently different courses in plasma and CSF. There were no significant changes of plasma concentration in the course. The CSF concentrations showed a continuous significant increase compared to the healthy controls: day 2 with *p* = 0.0074, day 4 with *p* = 0.0012, day 5 with *p* < 0.0001 (Kruskal–Wallis test) (Fig. 2E and F). The CSF-plasma index for IL-10 showed an increase on day 2 and a subsequent decrease. A characteristic differentiation with regard to the outcome was not possible due to missing values (Fig. 2G).

**Fig. 3:**
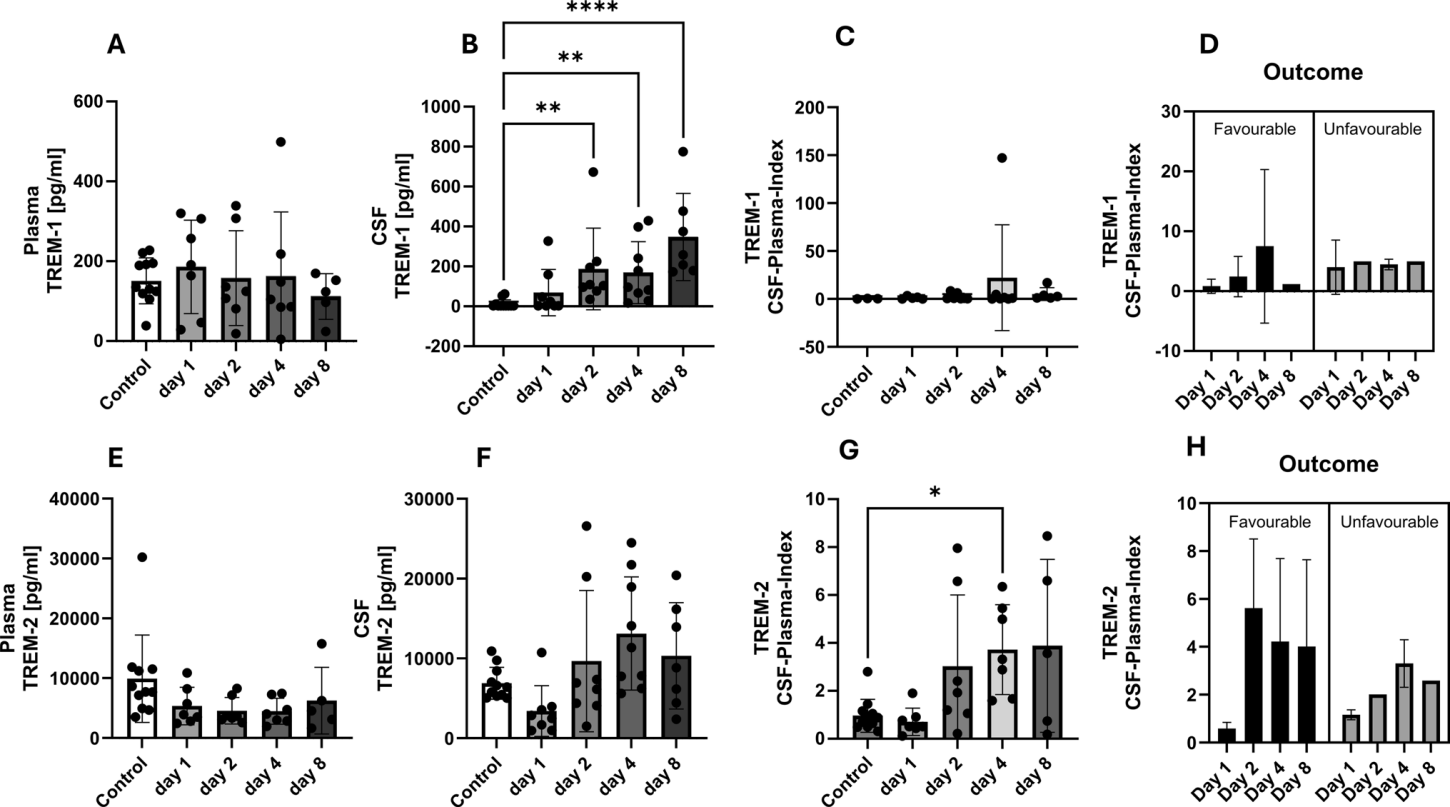
The plasma and cerebrospinal fluid (CSF) concentrations of TREM-1 and TREM-2 were measured over the course of the study, which spanned 8 days. The control patients each had an initial one-time value in the serum, and the CSF plasma quotient was used to assess the blood–brain (CSF) barrier function (quotient = CSF concentration / plasma (serum) concentration). TREM1 and TREM-2 appeared as late-stage biomarkers. Despite the observed variance in concentration over time between favourable and unfavourable outcomes, the potential for predictive power exists. However, it should be noted that significant results remain unattainable

Regarding the TREM1 and TREM2 balance, TREM1 concentrations in plasma remained approximately at the levels of control patients in serum whereas it increased with time in CSF peaking at the delayed injury phase. CSF concentrations of TREM1 increased significantly compared to control patients on day 2 (*p* = 0.004), on day 4 (*p* = 0.0055), and on day 8 (*p* < 0.0001, Kruskal–Wallis-Test) (Fig. 3A and B). The TREM-1 CSF-plasma index showed no characteristic course over time. Patients with a favourable outcome showed an increase in the CSF-plasma index with a decrease on day 8. Patients with an unfavourable outcome showed no characteristic course of the CSF-plasma index (Fig. 3C and D).

In contrast, TREM-2 plasma concentrations decreased below the levels of control patients and appeared unchanged for the further course. In CSF in the early phase (day 1), there was a decrease of TREM-2 concentration followed by an increase till day 4 (Fig. 3E and F). The TREM-2 CSF-plasma index revealed a significant difference between control patients and patients at day 4 (*p* = 0.022, Kruskal–Wallis test) (Fig. 3G). With regard to the outcome, there was a difference in the course of the TREM-2 CSF-plasma index. Patients with a favourable outcome showed a decrease of index from a higher level. Patients with an unfavourable outcome showed an increase of the index from a lower level (Fig. 3H).

**Fig. 4:**
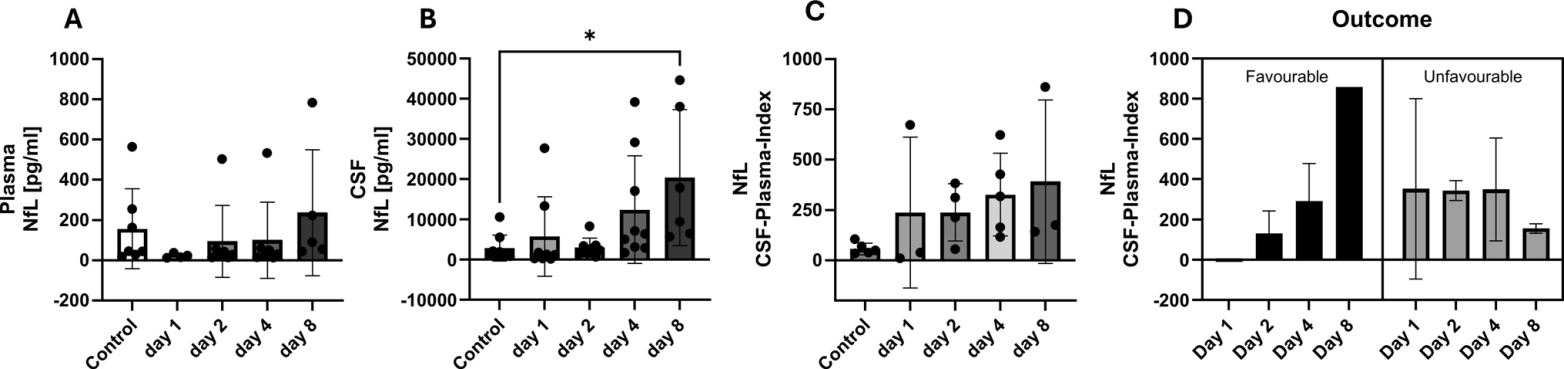
The plasma and cerebrospinal fluid (CSF) concentrations of neurofilament light chain over the course of the study, spanning 8 days. The control patients each have an initial one-time value in the serum, and the CSF plasma quotient is used to assess the blood–brain (CSF) barrier function (quotient = CSF concentration / plasma (serum) concentration). Neurofilament light chain appears as a biomarker of the late phase. Despite the observed variance in concentration over time between favourable and unfavourable outcomes, the potential for predictive power exists. However, there are no significant results for the outcome

For NfL, plasma levels increase from a concentration below that of the controls up to day 4 and exceed those of the healthy controls on day 8 (Fig. 4A). CSF shows a significant difference to healthy controls (*p* = 0.0104) when the values increase on day 8 (Fig. 4B). The CSF-plasma index shows elevated values compared to controls on all days, with an increase from day 2 to day 8 (Fig. 4C). Patients with a favourable outcome show an increase in the plasma CSF index, whereas there is no trend in patients with an unfavourable outcome (Fig. 4D).

**Fig. 5:**
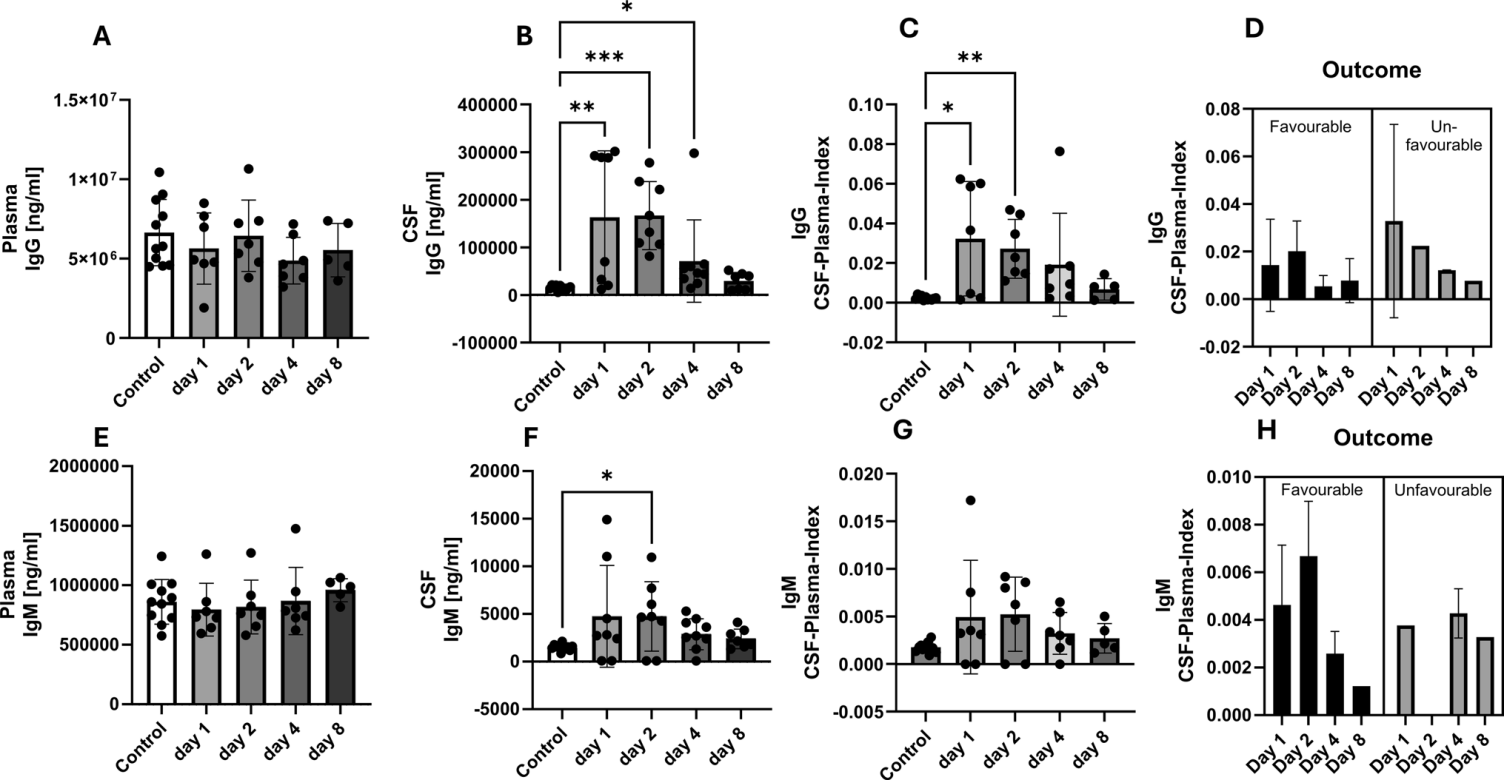
Plasma and cerebrospinal fluid (CSF) concentrations of IgG and IgM over the course of the study over 8 days. The control patients each have an initial one-time value in the serum. The CSF plasma quotient is used to assess the blood–brain (CSF) barrier function (quotient = CSF concentration / plasma (serum) concentration). IgG and IgM appear as biomarkers of the early phase. A clear distinction between patients with a favourable or unfavourable outcome cannot be derived from these biomarkers

Appearance of IgG in the CSF is reflecting a BBB dysfunction occurred during the early and intermediate phase after SAH while being unchanged in the plasma. Compared to the healthy controls, the CSF IgG concentrations showed a significant difference on day 1 (*p* = 0.004), day 2 (*p* = 0.0001) and day 4 (*p* = 0.0378, Kruskal–Wallis test) (Fig. 5A and B). Similar results were obtained for the IgG CSF-plasma index on day 1 (*p* = 0.0223) and day 2 (*p* = 0.0015, Kruskal–Wallis-Test) (Fig. 5C). A clear differentiation for the outcome could not be made on the basis of the IgG concentrations (Fig. 5D).

**Fig. 6:**
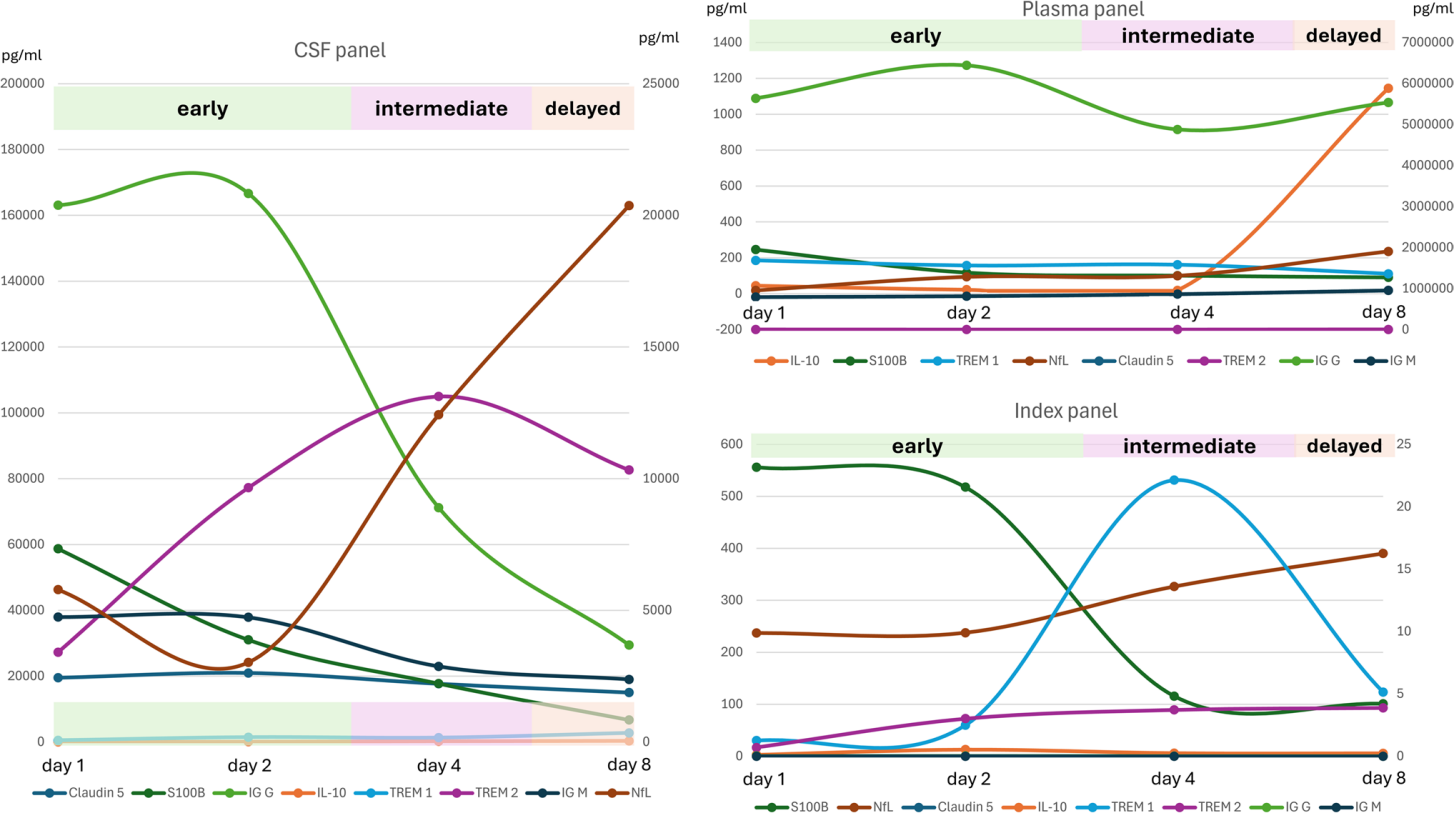
Combined concentration curves for the biomarkers S100B, Claudin-5, TREM-1, TREM-2, IL-10, IgG, IgM, neurofilament light chain (NfL) in the cerebrospinal fluid (CSF) and plasma as well as an index (CSF plasma quotient) representation. The CSF plasma quotient is used to assess the blood–CSF barrier function (quotient = CSF concentration/plasma (serum) concentration). Depending on the different compartments (CSF or plasma), the different biomarkers can be assigned different specific concentration curves and concentration peaks

A similar pattern was seen for IgM with a slight increase between day 1 and day 4 after SAH in CSF (day 2: *p* = 0.0279) and unchanged values in the plasma (Fig. 5E and F). The IgM CSF-plasma index is similar to the IgM CSF concentrations (Fig. 5G). A clear distinction of the outcome could not be made on the basis of the IgM CSF-plasma index courses either (Fig. 5H). Figure 6 shows the chronological order of the changes in concentration of the analysed biomarkers.

A differentiation with regard to the occurrence of vasospasms is not possible based on the available data (Supplement 1 A—E). The differentiation between patients with and without DCI showed different concentration curves of the TREM1 CSF-plasma index with increasing concentrations for patients with DCI (Supplement 1 G). The TREM 1, 2 and NfL CSF-plasma index shows higher concentrations for patients with DCI (Supplement 1 H-J). In patients with DCI, the IgG and IgM CSF-plasma index initially shows higher values, which decrease continuously over time. In patients without DCI, the CSF-plasma index concentrations tend to remain stable (Supplement 2).

## Discussion

The pathophysiological processes after SAH can be categorized into early, intermediate and late events [5]. During the period of early brain damage with endothelial dysfunction and neuroinflammation, the subsequent intermediate injury phase with dysfunction of the BBB, and the delayed injury phase marked by cellular changes, fundamental alterations in brain function may manifest clinically known as cerebral vasospasms and DCI [5, 67].

In our study, we were able to confirm S100B as a biomarker of the acute phase after SAH. Claudin-5 with its function of indicating the integrity and function of the BBB could be categorised into the intermediate injury phase after SAH. IL-10 showed a characteristic as a biomarker of the delayed injury phase, especially in CSF. On the fourth and eighth day, IL-10 showed the highest concentrations in the CSF, with the CSF-plasma index showing the highest concentrations on day 2. As with S100B and claudin-5, IL-10 could not be used to discriminate outcome or to differentiate patients with and without vasospasm or DCI with significant results. TREM-1 and TREM-2 with an important role in microglia-mediated inflammation and axonal injury showed concentration that can be categorised into the delayed injury phase. Due to different concentration curves of the CSF-plasma index for TREM-1 and TREM-2, a differentiation between favourable and unfavourable outcome seems possible. The same applies to the differentiation between patients with and without vasospasm and DCI, although no significant effects were found. Nfl also tended to be a biomarker of the delayed phase with an increase towards the eighth day. Patients with a favourable outcome showed an increase on the eighth day. IgM and IgG appear with their early increases in concentration on days 1 to 4 as biomarkers of the early injury phase.

When examining the CSF and PB of the clinical SAH setting, a distinction must be made between the intentions of making the diagnosis on the one hand, and the classification of injury severity, identification of patients at risk, differentiation of treatment concepts and prognosis on the other hand [68, 69]. The critical care course with endovascular and surgical interventions, long-term access to vessels, the requirement of artificial ventilation and CSF diversion renders the differentiation between CSF changes after SAH and infections in the CSF space rather difficult. Whereas SAH causes an increase in leukocytes, chloride and glucose in the CSF [70], the most common CSF signs of a bacterial infection are decreased glucose concentrations, elevated protein levels and increased white blood cell count [71]. Pathogenic infection of CSF could be ruled out by regularly biochemistry and microbiological monitoring. However, these parameters represent only a small diagnostic window into a rather complex immuno-pathophysiological response to SAH.

Our selected panel of biomarkers attempts to reflect the previously summarized pathophysiological processes. Specifically, the phase of early brain injury is assigned to S100B, TREM1 and TREM2 (microglia-mediated inflammation), the intermediate phase is assigned to Claudin-5 (BBB breakdown) and the delayed injury phase is assigned to IL10 and NfL. Noteworthy, the IgG/IgM and Claudin-5 profiles indicate some BBB changes in the early and intermediate phase, all of which was cleared at day 8 after haemorrhage, indicating resealing of the BBB. In contrast, TREM-1 in CSF increased especially in the delayed phase, which could reflect some infectious/inflammatory problems. In the context of stroke modelled by occlusion of the middle cerebral artery, an elegant study by Liu et al. demonstrated that brain damage resulted in TREM-1 upregulation on peripheral myeloid cells 48 h after occlusion including enhanced TREM concentrations. TREM-1 seems to be critically involved in activation of peripheral innate immunity resulting in recruitment of inflammatory cells to the brain after stroke and in parallel also in inflammatory damage of the gut-blood-barrier allowing bacterial translocation and infectious complications [72, 73]. In an experimental approach of SAH, Sun et al. showed an early increase in TREM-1 concentration in the brain after 6 h and a peak after 48 h. The activation was attributed to microglia and endothelial cells. TREM-1 inhibition showed an attenuation of early brain injury as measured by TLR4, MyD88, NF-kB suppression [74]. The same group was able to show in 34 SAH patients that the concentration of TREM-1 increases with the Hunt & Hess grade (I-II, III, IV-V), correlates statistically significantly and correlated negatively with the GCS [75, 76]. However, patients in their study also underwent aneurysm surgery. The interpretation of the results is therefore not easy, as it is not possible to differentiate between SAH-initiated or surgically induced inflammation. Thus, it is tempting to speculate that the late TREM-1 increase in CSF might reflect some challenges to innate immunity within the brain, likely caused by cellular debris or invading microbe-associated molecular patterns (MAMPs). In contrast, TREM-2 seems rather to act anti-inflammatory [72, 73], which accordingly in our cohort was unaltered if not decreased in peripheral blood. Similarly, IL-10 as mainly anti-inflammatory mediator was significantly upregulated in the liquor of several patients (but not systemically) at the delayed injury phase, possibly reflecting the spatial efforts of inflammatory resolution within the brain. NfL was published repeatedly and mostly not in the CSF in connection with the delayed phase after SAH [77–80]. However, overall, the temporal allocation cannot be clearly separated. The different pathophysiological phases merge into another and are subject to individual influences.

Numerous cytokines, interleukins and mediators in PB, ventricular and lumbar CSF have been investigated in the treatment of SAH. Indices were created to better visualise intra- and intercellular processes [81–83]. Differentiations were made with regard to the severity [84–90], the outcome [84–87, 89–100], the occurrence of vasospasms (22, 101–106) and the DCI [85–87, 93, 104, 106–113]. Biomarkers were also identified to differentiate between pathophysiological processes after SAH and nosocomial infections [114] or patients with and without aneurysms in spontaneous SAH [115].

Cerebral vasospasm in connection with DCI is a predictor of morbidity and mortality [1, 116]. Even if the treatment of vasospasms alone has only a limited influence on the outcome after SAH, the early and reliable detection of vasospasms is responsible for risk stratification, initiation of prophylactic measures and the early detection and treatment of DCI [1]. Our panel of biomarkers was not able to differentiate significantly between patients with and without vasospasm. Przybycien-Szymanska and Ashley summarized genetic marker (haptoglobin 2–2 gene polymorphism, ApoEε4 gene polymorphism, 219 T ApoE promoter polymorphism), cell damage marker (Neuron-specific enolase, UCHL1, CCSctf, 14–3-3ß, pNF-H, SBDP), inflammation markers (c-reactive protein), energy metabolism markers (glutamate, glutamine, histidine, glycine, ApoE), vascular tone markers (endothelin-1, calcium) and microparticle markers (CD142 tissue factor, CD41 platelets, CD235a erythrocytes, CD146 endothelial cells, CD66b neutrophils, von Willebrand Factor labeled microparticles, CD105 labeled endothelial microparticles) for an increased risk of vasospasm after SAH [22]. Lad et al. identified a connection and summarized endothelin-1, Interleukin 6, E-selectin, Nox, lactate, SBDPs, ADMA, PDGF, adrenomedullin and the thrombin activity with the occurrence of symptomatic, radiologic vasospasm or a coincidence with vasospasm [101]. Interleukin 6 in particular was analysed as a biomarker for the presence of vasospasm [103, 106]. Nevertheless, the exact pathophysiological classification of the occurrence of angiographic, symptomatic or DCI-related vasospamus remains unclear. So far, only a few pathophysiological steps have been described using biomarkers such as neuropeptide Y [104] or Alpha-II spectrin breakdown products [105] as representatives for the early brain injury. It is the opinion of the authors that no biomarker or panel of biomarkers currently exists that can indicate the risk of symptomatic and/or radiological vasospasm with high sensitivity and specificity in the early phase or monitor treatment success in the intermediate phase.

After the initial haemorrhage, DCI has the greatest contribution to unfavourable outcome after SAH [117]. In the context of SAH, various microRNAs have been identified for the early injury phase [86]. Based on the overexpression of various microRNAs, a distinction could be made between patients with and without DCI. However, the results were not always significant [102, 118, 119]. Wang et al. described a linear relationship between serum sestrin 2 concentrations and the risk of DCI, although this result was not significant. Sestrin 2, which is mainly expressed in the CNS by neurons and microglia, is thought to have neuroprotective functions in the context of oxidative, hypoxic, inflammatory or ischaemic stress [85, 120]. Especially, the oxidative stress that is formed in cerebrovascular endothelial cells and neurons, for example, by nicotinamide adenine dinucleotide phosphate oxidases (NOXs) with the subgroup NOX4 [121] after SAH [122]. Pan et al. found in their cohort that patients with a serum NOX4 concentration of ≥ 11 ng/ml have a significantly increased risk of DCI [87]. In the context of hypoxia-induced stress, hypoxia-inducible factor-1 (HIF-1), an oxygen-sensitive transcription activator, showed significantly higher serum levels in patients with DCI compared to patients without DCI [93, 123]. Serum HIF-1 alpha concentrations > 229.3 pg/ml predict the presence of DCI with medium–high sensitivity and specificity. In the context of inflammatory cascades after SAH and the pathophysiological processes leading to DCI, Croci et al. and Simon et al. have attributed an important role to the cytokine interleukin 6 as a biomarker, which is increased in patients with DCI [106, 111]. Nevertheless, there are still considerable gaps in our knowledge of cytokine pathways. Savarraj et al. described the potential role of leucine-rich-alpha-2-glycoprotein 1 (LRG1) as modulator in the endothelial transforming growth factor-ß (TGFß) signalling pathway and DCI biomarker [107, 124]. In their study, patients with DCI revealed significantly higher plasma concentrations than patients without DCI; however, this could not be reproduced for TGF1ß und TGF2ß [107]. The same appeared to the enzyme dipeptidyl peptidase 3 (DPP3), which was increased in critically ill patients and showed to be a predictor for DCI in SAH patients [109]. This non-exhaustive list illustrates the many different approaches in finding reliable biomarkers for identifying the risk of DCI and the complexity of the pathophysiological processes that can only be partially reflected by the biomarkers mentioned.

In the 1990s and early 2000s, research on outcomes after SAH was dominated by assessing the influence of patients or clinical factors such as sex, age, initial severity of haemorrhage or diagnostic imaging results [125]. However, despite guidelines, treatment was often subject to different measures, such as different times of different diagnostic imaging procedures, which resulted in different prophylactic, therapeutic or metaphylactic measures [125]. The evaluation of the outcome after SAH continues to be very difficult as many different influences, such as clinical factors (clinical and image guided grading of the severity of the haemorrhage), the course with the occurrence of complications such as DCI or the various options for determining the outcome, have an influence on this [126]. And despite well-developed guidelines, patients’ individual courses of treatment are sometimes characterised by individual decisions made. Due to the great variability of clinical and imaging factors, biomarkers have been proposed as a reflection of the actual biological and pathophysiological processes involved in predicting outcome [125]. Apart from the biomarkers Sestrin2, MicroRNA, NOX4 already mentioned which influence the outcome via the occurrence of the DCI, the biomarkers tumour necrosis factor α-inducible protein 3 (A20) are of relevance [84].

There are also some limitations of the present descriptive study. The number of patients was limited and thus statistical significance might be underestimated and thereby some clinical correlations missed. The low number of patients in the period described is due to the combination of very high patient workload (sample collection, processing and clinical examinations every 6 h over a maximum of 12 days) and the lack of trained specialist staff. In future studies, sufficient financial resources should be secured to involve more specialised personnel and thus increase the number of patients included. Especially towards the end of the study or towards day 11, it was possible that not all samples could be obtained, e.g. one patient died or the processing did not yield any usable results. The relative sample density was indicated in the graphs as point values. Due to the small number of patients, statistical methods such as multiple imputation could not be applied. Due to the small number of patients and the lack of options to replace missing values, the results can only be indicative, should be confirmed in a larger, multicentre study and should not be generalised. Another limitation is the different blood matrices between patients and the control population. In further studies, the methods should be the same for both populations in order to maximise comparability. There are also some novel biomarkers such as α-synuclein as synaptic damage maker, which have been applied in the traumatic brain injury setting with a high predictive performance [127] but are less used in the context of SAH. It seems almost impossible to do justice to the constantly growing number of biomarkers and to mention them all. Further profiling will help closer define the temporo-spatial response to SAH.

## Conclusions

Despite the continuous improvement in the identification of SAH patients, aneurysm occlusion methods and clinical treatment, including critical care measures, pathophysiological knowledge of the secondary injury phase remains limited. Consequently, the clinical possibilities of identifying patients at risk for relevant vasospasms or DCI remain limited. Numerous biomarkers have been proclaimed in the pathological condition of SAH, but to the best of our knowledge there are still no biomarkers or a combination of biomarkers that can predict the course of treatment or outcome with high specificity and sensitivity. It seems sensible to combine efforts and investigate known biomarkers with regard to their predictive potential using a panel and a large number of patients.

## Supplementary Information

Below is the link to the electronic supplementary material.

ESM 1 (PNG 102 KB)

High Resolution Image (TIF 145 KB)

ESM 2 (PNG 54.4 KB)

High Resolution Image (TIF 88.1 KB)

ESM 3 (DOCX 14.4 KB)

## Data Availability

Raw data are available from the corresponding author by request. This regulation was chosen on the basis of case law in Germany, as patient-specific data could be used to identify patients with great effort.

## Abbreviations

BBB: Blood-brain barrier
BCS: Brussels Coma Scale
CNS: Central nervous system
CRP: C-reactive protein
CSF: Cerebro-spinal-fluid
CT: Computed tomography
DCI: Delayed cerebral ischaemia
DSA: Digital subtraction angiography
ELISA: Enzyme-linked immunosorbent assay
GCS: Glasgow Coma Score
GOS: Glasgow Outcome Scale
Ig: Immunoglobulin
IL: Interleukin
MAMP: Microbe-associated molecular patterns
NfL: Neurofilament light chain
PB: Peripheral blood
PTT: Partial thromboplastin time
SAH: Aneurysmatic subarachnoid haemorrhage
SD: Standard deviation
TBI: Traumatic brain injury
TCD: Transcranial Doppler ultrasound
TREM: Triggering receptor expresses on myeloid cells
