# Supplementary figures and images for: Biomarkers in Aneurysmatic and Spontaneous Subarachnoid Haemorrhage: A Clinical Prospective Multicentre Biomarker Panel Study of S100B, Claudin-5, Interleukin-10, TREM-1, TREM-2 and Neurofilament Light Chain As Well As Immunoglobulin G and M
Source: Mol Neurobiol. 2025 Apr 28;62(9):11499–516. doi: 10.1007/s12035-025-04889-3 (PMC12367908; doi:10.1007/s12035-025-04889-3)

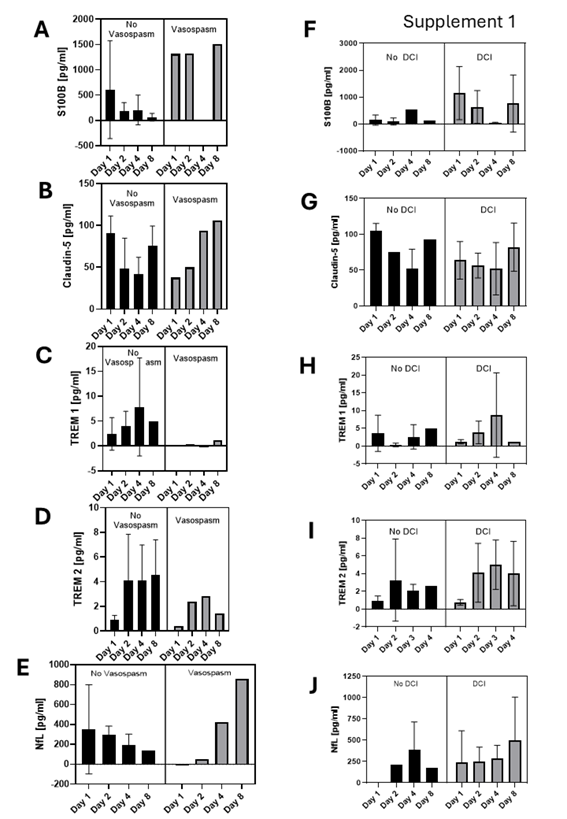

Supplement: Supplementary file 1 — (PNG 102 KB) [file 12035_2025_4889_Fig7_ESM.png]

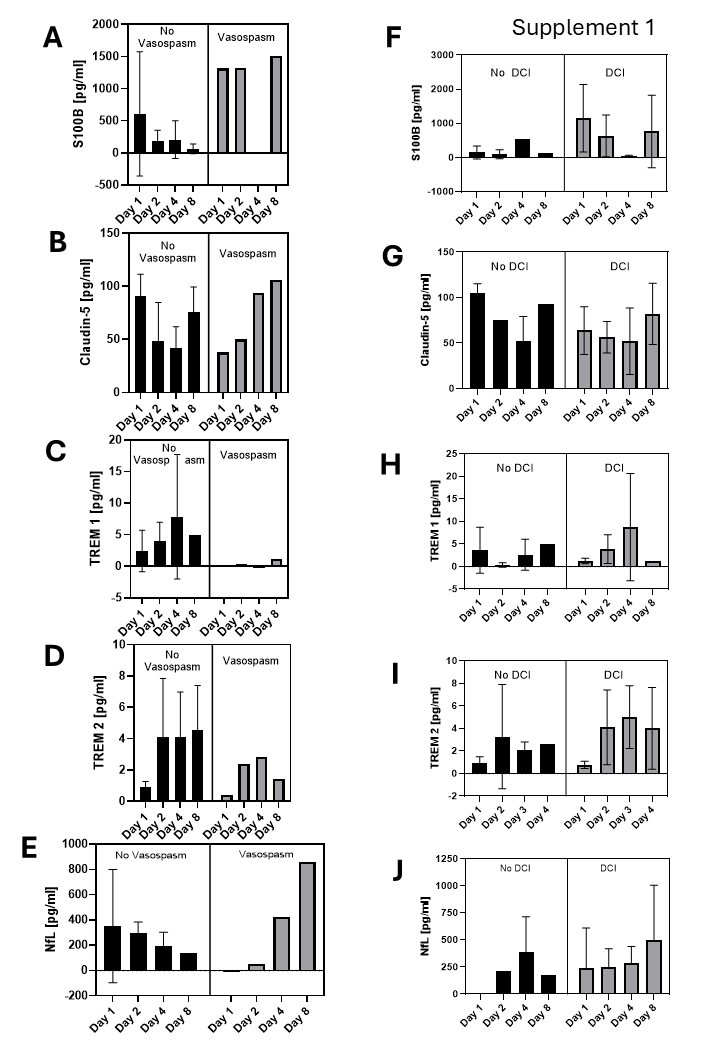

Supplement: Supplementary file 2 — High Resolution Image (TIF 145 KB) [file 12035_2025_4889_MOESM1_ESM.tif]

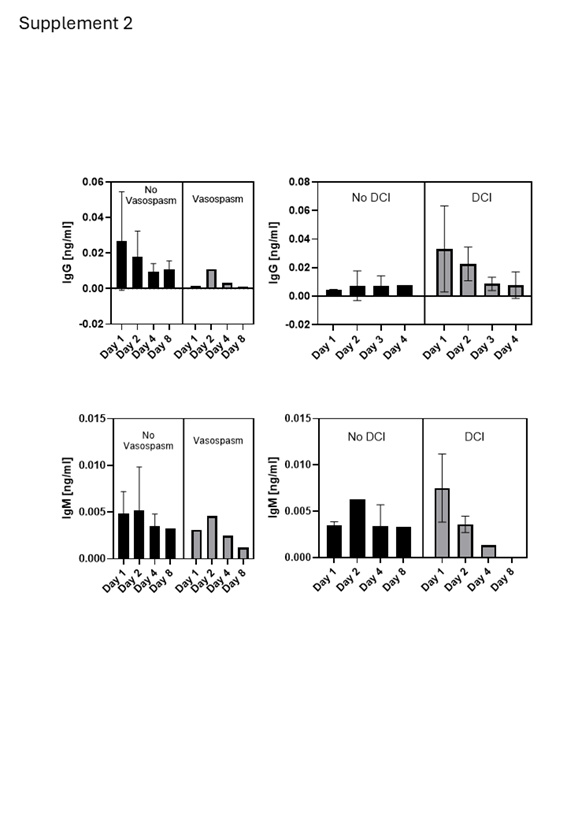

Supplement: Supplementary file 3 — (PNG 54.4 KB) [file 12035_2025_4889_Fig8_ESM.png]

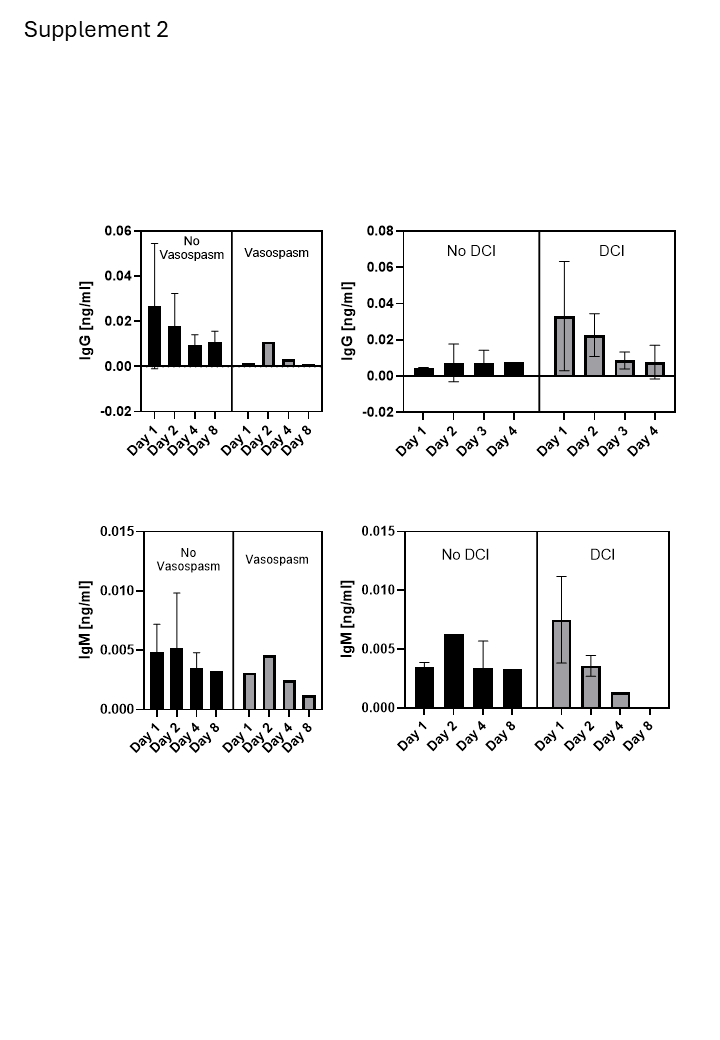

Supplement: Supplementary file 4 — High Resolution Image (TIF 88.1 KB) [file 12035_2025_4889_MOESM2_ESM.tif]
