# Supplementary material for: Biomarkers in Aneurysmatic and Spontaneous Subarachnoid Haemorrhage: A Clinical Prospective Multicentre Biomarker Panel Study of S100B, Claudin-5, Interleukin-10, TREM-1, TREM-2 and Neurofilament Light Chain As Well As Immunoglobulin G and M
Source: Mol Neurobiol. 2025 Apr 28;62(9):11499–516. doi: 10.1007/s12035-025-04889-3 (PMC12367908; doi:10.1007/s12035-025-04889-3)
Supplement: Supplementary file 5 — (DOCX 14.4 KB) [file 12035_2025_4889_MOESM3_ESM.docx]

Supplement table 1

| **Diagnoses of the control patients** |
| --- |
| Gait uncertainty (clinical) |
| Left basal ganglia damage (metabolomic) |
| Polyneuropathy of unknown origin |
| Anterior ischemic optic neuropathy |
| Depression |
| Myasthenia gravis |
| Convulsive syncope |
| Facial palsy (idiopathic) |
| Facial palsy (idiopathic) |
| Tension headache |
| Tension headache |
